# The performance of mind: from movement, mental states, and consciousness

**DOI:** 10.3389/fpsyg.2025.1736933

**Published:** 2026-01-12

**Authors:** Guy Cheron, Ana Maria Cebolla

**Affiliations:** 1 Movement Neurophysiology Lab NeuroMove, ULB Neuroscience Institute, Université Libre de Bruxelles, Brussels, Belgium; 2 Neurosciences Laboratory, University of Mons (UMONS), Mons, Belgium

**Keywords:** brain-dynamics, consciousness, EEG, mental state, movement, neural integrator, performance

## Abstract

Integrative neuroscience increasingly recognizes that the brain evolved primarily as a biological system for generating movement. Viewed as a complex oscillator, the brain is now widely investigated through electroencephalography (EEG), which occupies a central position in both motor neuroscience and cognitive research, particularly in the study of consciousness. In this perspective article, we revisit experimental findings from both animal models and humans demonstrating how the analysis of brain oscillatory dynamics including neural entrainment allows the investigation of mental states, motor performance, and consciousness. By examining three well-established electrophysiological markers (the P300 evoked potential, the readiness potential, and the somatosensory N30 wave), we propose that new neurophysiological mechanisms may be identified and explored through future experimentation. We further suggest that insights from oculomotor research, especially the concept of the neural integrator and its extension to working memory and dynamic attractor models, may help clarify the functional interplay between movement generation and consciousness.

## 1 Introduction

This perspective article seeks to advance a comprehensive vision of the movement sciences by adopting an integrative framework that places motor action at the core of empirical investigation (Gramann et al., 2011, 2014; Leroy and Cheron, 2020). This approach highlights the continuous interplay between the neurophysiological mechanisms underlying movement and their extensions into cognitive and affective domains (Vitkova et al., 2024; Hashemi et al., 2025).

Building on this view, we advocate for an empirical framework that integrates the analysis of brain electrical activity across diverse mental states, including investigations of consciousness, during the execution and control of motor actions. This framework is grounded in both theoretical and experimental insights derived from research on ocular motricity.

## 2 The brain’s oscillatory dynamics

Brain physiology cannot be reduced to the electrical activity of neuronal populations alone; it also relies on biochemical processes and neuronal plasticity involving genetic signaling and protein synthesis (Kandel, 2001). Nevertheless, the brain can be viewed as a complex oscillator (Buzsaki, 2011), and electroencephalography (EEG) provides valuable insights into brain function (Cheron et al., 2016b) and dysfunction (Zarka et al., 2020, 2021; Zhang et al., 2025). Although EEG is one of the most widely used tools in neurology and psychiatry, few fundamental studies have clarified the precise neurophysiological origins of its signals. Simplistic interpretations, such as global “excitation” or “inhibition” of the brain, often obscure the underlying complexity of synaptic and network processes. A deeper understanding will require interdisciplinary and experimental integration.

The standard model attributes EEG signals to the summation of postsynaptic potentials within neuronal populations. Yet, as Cohen (2017) notes, this view remains incomplete, and further research is needed to uncover the mechanisms governing these scalp-recorded signals. Previous work has sought to bridge human EEG studies and animal models (Cheron et al., 2016b). Combining patch-clamp recordings and local field potentials (LFPs) in the somatosensory cortex of mouse the Petersen’s group (Poulet and Petersen, 2008; Poulet et al., 2012; Petersen, 2017) has provided cross-species insights into neural rhythms linking the membrane synchrony of the recorded neurons, the firing behavior, the LFP and the EEG. Significant progress achieved in understanding human diseases through mouse-based approaches (Cheron et al., 2005; Hourez et al., 2011; Prigogine et al., 2024) encourages us to continue pursuing parallel investigations in both species as recommended by the impressive review of the critical rhythms by Wang (2010).

EEG oscillations reflect rhythmic fluctuations in neuronal activity that organize cognition, perception, and behavior. Theta oscillations (4–8 Hz) are the most functionally versatile, followed by beta (15–30 Hz), alpha (8–15 Hz), gamma (30–150 Hz), and delta (0–4 Hz) rhythms (Cheron et al., 2016b). This does not imply hierarchy, but rather the adaptability of oscillatory dynamics across mental states. For instance, alpha waves propagate from higher order to primary cortical and subcortical regions during wakefulness (Halgren et al., 2019) but form rotating waves during sleep (Muller et al., 2018). These studies reveal a functional geometry of the brain emerging from its global oscillatory organization.

One of the major advantages of oscillatory patterns is that they allow at least two key non-redundant mechanisms to be investigated: amplitude modulation and phase modulation. Increases in the neuronal population recruitment and their relative synchrony with respect to external or internal events can be examined through event-related spectral perturbation (ERSP) and inter-trial coherency (ITC) (Makeig et al., 2002). The analysis of phase-locking within specific frequency bands paved the way for the Communication-Through-Coherence (CTC) hypothesis (Fries, 2005, 2015), which proposes that phase alignment across oscillations facilitates the formation of selective neural pathways, thereby promoting cognitive flexibility. Closely related to CTC, the Dynamic Information Selection by Entrainment (DISE) theory (Lakatos et al., 2019) conceptualizes entrainment as a physical mechanism that establishes stable relationships not only between distinct neural oscillations, but also between neural rhythms and independent external or motor rhythmic patterns (Lakatos et al., 2019; Schmid, 2024). As we will see later, such neural entrainment may explain many properties of evoked potential responses.

## 3 Mental states and performance

The achievement of a performance, whether motor or mental, depends on the mental state that precedes and accompanies it. Brain states [e.g., wakefulness with low or high arousal, anesthesia, slow wave sleep (SWS), and rapid eye movement (REM)] have been associated with behavioral states. However, they are not homogeneous static entities only characterized from synchronized, or desynchronized neuronal activity but may involve complex forms of microstates transitory brain dynamics (Olcese et al., 2018). The brain’s baseline activity, representing a true multiplexing of the nervous system’s various needs required to maintain the organism’s homeostasis, also determines this mental state. In this line, McCormick et al. (2020) proposed a complex dialogue between the behavioral state and the dynamics of the mental state expressed by a point in a high-dimensional phase space representing the neural activity of different brain structures. This point moves over time through this dynamic space along a trajectory unique to everyone. Individual specificity may thus explain the difficulty of finding common activation pattern coding among subjects. Only relational coding would achieve inter-subject consistency in visual recognition task (Lipman et al., 2025). Individual dynamics is, in a sense, holistic, requiring us to consider the full set of behavioral (motor, sensory, visceral) and psychological variables, while being fundamentally centered on individual brain approach.

This conception aligns with the objectives we pursue in our *Frontiers* section, which seeks to promote integrated neuroscience research in which different behavioral states, those related to basic physiology such as cardiac and respiratory activity, as well as body and eye movements, can be correlated with mental states. The detection of mental states can be facilitated by measuring pupil diameter, which may be coupled with eye movement recordings and EEG. Using this approach, David et al. (2025) demonstrated a reduction in pupillary response in Alzheimer’s patients who showed morphological alterations of the locus coeruleus on MRI, as well as a correlation between changes in pupil diameter during the presentation of salient stimuli and the slowing of the alpha rhythm. According to the psychophysical law of Yerkes and Dodson (1908) optimal performance occurs, following an inverted U-shaped curve, at a moderate level of cortical excitability. This mental state can be investigated through measurements of pupil diameter fluctuations, which are influenced by neuromodulators such as catecholamines (dopamine and noradrenaline). For example, atomoxetine, a noradrenaline reuptake inhibitor, increases arousal and shifts the Yerkes–Dodson curve while a cholinergic agent donepezil has not significant arousal effect on the curve (Beerendonk et al., 2025; de Gee et al., 2025). However, it is important to remain cautious because individual factors and different methodological approaches might explain variations in this type of relationship in motor performance.

## 4 The labyrinth of consciousness research

In the evolution of brain sciences devoted to consciousness, two main approaches have diverged: neuropsychological studies of consciousness and fundamental neuroscience exploring neuronal network dynamics down to dendritic, molecular, and genetic mechanisms. A major challenge lies in distinguishing between wakefulness—our ability to perceive external stimuli and actions—and phenomenal consciousness, the subjective awareness of being aware (Brown et al., 2019). This distinction separates the concept of consciousness from the sense of self that disappears during sleep and reemerges upon awakening.

The debate intensifies with the Higher-Order Theory (HOT) of consciousness (Rosenthal, 1998, 2012), which posits that higher-order representations are necessary for phenomenal consciousness. Initially philosophical, this model gained neuroscientific support through Lau and Rosenthal (2011). In contrast, the Global Workspace Theory (GWT) (Dehaene et al., 1998; Dehaene and Naccache, 2001) offers a competing view, fueling a two-decade-long debate shaping the field.

Such theoretical contrasts underscore the need for empirical methods capable of capturing large-scale brain dynamics in real time. Electroencephalography (EEG), with its high temporal resolution, remains particularly suited to investigating how conscious states and their dissociations emerge from ongoing neural activity.

## 5 Movement and consciousness

While the distinction between the “easy” and “hard” problems of consciousness (Chalmers, 1998) has shaped philosophical debates (Seth and Bayne, 2022), neuroscience increasingly highlights the need for empirical approaches grounded in measurable brain dynamics. EEG, in particular, offers a unique opportunity to study large-scale neural activity not only at rest but also during movement and complex behaviors. By linking oscillatory patterns to locomotion, sport, and motor interactions, this approach bridges subjective experience with objective recording. In doing so, it opens new avenues for understanding how consciousness is embedded in action (Cebolla and Cheron, 2019), and how brain–body coupling contributes to both basic states and higher-order awareness.

If a mental state is considered as a dynamic structure, how can a meta-consciousness also conceived as a dynamic state—be understood independently from consciousness itself, which is likewise the result of a dynamic structure? In such a case, it would need to be demonstrated that these two dynamic states are truly independent of one another, something that has yet to be formally established. Even though neurology describes very specific situations in which a patient is awake and performs seemingly normal movements while failing to respond to any instructions appearing unconscious of both their environment and their actions (Damasio, 2000) is highly likely that the epileptic disorder underlying such behavior specifically affects vulnerable points within a broad dynamic network. This would produce a state of dissociation without necessarily implying a functional dichotomy between two distinct types of consciousness.

It was Caton (1887) who first proposed that patterns of electrical brain activity depend on the animal’s behavioral state. This notion is now expressed in terms of the mental state, which has been characterized down to the level of neuronal membranes and synaptic transmission processes in which oscillatory entrainment may also occurred (Llinas, 1988; Crochet and Petersen, 2006; Poulet and Petersen, 2008; Poulet et al., 2012). Approaching the science of consciousness by integrating experimental findings from both animal preparations and humans may represent another path toward the development of an integrative neuroscience. Such an approach could foster the emergence of new experiments grounded in empirical evidence across multiple levels of organization (Cheron et al., 2016a) from genes, neurons, and networks to behavior and mental states.

Seth and Bayne (2022) identified 22 distinct theories of consciousness grounded in neurobiological evidence. In addition, several hybrid accounts emphasizing factors such as attention, learning, recurrent loops, emotional regulation, and subcortical mechanisms further illustrate the diversity and complexity of current theoretical approaches. This profusion of diverse theories nevertheless raises certain questions. Are they all fundamentally different, or do they result from an intellectual play with words and psycho-philosophical concepts that remain somewhat distant from phenomenology and, above all, from the empirical facts derived from the scientific method?

The privileged link between movement and consciousness becomes fully meaningful when considering the concepts of brain–body coupling and consciousness (Tallon-Baudry et al., 2018; Azzalini et al., 2021) embedded in action through oscillatory entrainment. These research themes should be further developed, as they are likely to underlie the continuous interactions between what are traditionally referred to as the “easy” and “hard” problems of consciousness. It has become also essential to revisit older experimental findings, reanalyze them using modern technologies, and then confront them with this multiplicity of theories. One of the latest trends in this respect is the development of so-called adversarial collaborations (Melloni et al., 2023; Cogitate Consortium et al., 2025; Naccache et al., 2025), bringing together different groups of researchers who are supportive of certain theories, while avoiding more radical confrontations with other theories.

Consider, for instance, a few simple experiments such as the P300 component of event-related potentials (Chapman and Bragdon, 1964; Desmedt et al., 1965; Sutton et al., 1965), the readiness potential preceding voluntary action (Deecke et al., 1969; Libet et al., 1983; Fried et al., 2011), and the mental gating of the N30 component of somatosensory evoked potentials (Cheron and Borenstein, 1992). What do these evoked potentials tell us about consciousness?

### 5.1 The P300 wave as the index of sensory consciousness

The P300 wave may lie at the crossroads between sensory consciousness and unconscious processing, as it reflects the emergence of a globally evoked component of brain electrical activity when we become aware of a sensory stimulus, whether somatosensory, auditory, or visual. Conversely, this implies that before approximately 300 ms, brain activity occurs unconsciously. In other words, earlier evoked responses, although remaining unconscious, can still exhibit temporal, frequency, and spatial variations depending on different states of consciousness.

EEG dynamic analysis of these unconsciously generated activities may therefore reveal anticipatory processing occurring before 300 ms, potentially leading to the emergence of sensory consciousness. The bifurcation observed around 270 ms in the attentional blink experiment by Sergent et al. (2005) provides a good example of this process, showing a progressive trend toward the conscious recognition of a sensory event.

Since the early P1 and N1 components are also evoked by unseen visual stimuli, their modulation represents a form of unconscious feature analysis, as previously described for somatosensory stimuli (Desmedt and Robertson, 1977), which later contributes to the conscious recognition reflected by the P300 wave. The identification of the corresponding brain generators during the attentional blink experiment (Sergent et al., 2005) also revealed a progressive spread of activity across a distributed network of cortical association areas, consistent with the Global Neuronal Workspace model (Dehaene et al., 1998). The early unconscious evoked components can also be interpreted, in accordance with the CTC (Fries, 2005) and DISE (Lakatos et al., 2019) models, as reflecting oscillatory phase-locking and entrainment that support the processing of sensory features. This process leads to a bifurcation point (Klatzmann et al., 2025) at which a specific neural entrainment gives rise to the subsequent P300 component associated with the conscious perception of the sensory event.

### 5.2 The readiness potential

The negative potential preceding voluntary action, as revealed by Libet’s seminal experiment (Libet et al., 1983), opened the debate on the neural basis of free will (Triggiani et al., 2023; Blagovechtchenski et al., 2025; Verbaarschot et al., 2025). The controversy stems from the finding that the subject’s awareness of the moment they decide to perform a motor act (such as finger flexion) occurs several hundred milliseconds after the onset of the readiness potential. This observation has been widely interpreted as suggesting that the initiation of voluntary action begins at an unconscious level. However, less attention has been given to the fact that the conscious *intention* to move the finger was established before the onset of this negative potential (Desmurget et al., 2009; Desmurget and Sirigu, 2009). What appears to be delayed is not the decision itself, but rather the conscious awareness of the *exact timing* of movement initiation. Indeed, in Libet’s experiment, participants had already made a conscious decision to execute the final action. Consequently, it is through a consciously guided process that neurons across multiple cortical (Fried et al., 2011; Fried, 2022) and subcortical regions are progressively recruited in a ramp-like manner (Emmons et al., 2017). Through this gradual accumulation of neural activity (accumulator model) (Schurger et al., 2012) the threshold for movement initiation is ultimately reached at a specific timing, one that we become aware of, but only with a temporal delay. The timing and amplitude of the readiness potential can also be modulated during learning (Jochumsen et al., 2017). Other nonlinear dynamic mechanisms might account for the initiation and maintenance of the readiness potential (Moutard et al., 2015). Based on recurrent loop mechanisms supporting cascades of integrators, a neural ignition (Fisch et al., 2009) threshold can dynamically switch the system from the spontaneous resting-state mode of ongoing stochastic activity to an active mode. This neural “ignition” could thus initiate the readiness potential as well as the associated content-specific awareness.

### 5.3 The mental gating of the N30 somatosensory component

Electrical stimulation of the median nerve give rise to well described evoked potentials among those the N30 wave (Desmedt and Cheron, 1980) occurring about 10 ms after the afferent volley reaches the primary somatosensory cortex, as indexed by the N20 wave have attracted attention because of their physiological modulation induced by sensori-motor and cognitive activities (Cheron, 1999; Cebolla and Cheron, 2015). For instance, the N30 wave can be suppressed or even inverted when one imagines moving their fingers (Cheron and Borenstein, 1992). This places us in a very early temporal domain, well before conscious awareness of peripheral electrical stimulation. However, a change in mental state, specifically, the execution of a cognitive-motor imagery task that Roland et al. (1980) showed to depend on SMA activation was able to suppress an early component of somatosensory evoked potentials.

At the time, these electrophysiological phenomena were interpreted as a form of gating or interference between a conscious mental state differing from rest and the brain’s unconscious processing of a sensory signal. Since the introduction of the top-down framework (Engel et al., 2001), increasing evidence has shown that top-down signals modulate the intrinsic dynamics of thalamocortical networks, shaping predictions about forthcoming sensory input. Within this perspective, the suppression of the N30 component may be interpreted as the outcome of top-down influences acting upon the N30 generators—distributed across a network including the precentral gyrus (BA4), the supplementary motor area (BA6), and BA9—and supported by beta-gamma synchronous oscillations (Cheron et al., 2007; Cebolla et al., 2009, 2011). This is indeed the alteration of these synchronous oscillations that could explain the modulation of the N30 (Cebolla et al., 2009). Similarly, when the stimulated hand is observed, the direction of gaze induces a specific increase in N30 amplitude (Rossi et al., 2002; Cebolla et al., 2014), demonstrating the power of a conscious task on very early electrophysiological processes. Examined under the DISE theory (Lakatos et al., 2019), the pure phase-locking of the N30 at rest and its amplification when visual attention was directed to the stimulated hand can be explained by an oscillatory entrainment indexed by alpha and beta-gamma phase-locked by the concomitant visual flux (Cebolla et al., 2014). The fact that about 70% of the trials, indexed by ITC analysis, given rise to the N30 wave corresponds to a pure phase locking of beta-gamma oscillations (Cheron et al., 2007) opens the fundamental debate about the physiological mechanism supporting the rapid phase locking change at this short latency. *In vitro* experiments demonstrated the role played by the gap junction for the induction of rapid switch of the oscillatory phase in response to blocking of gap junction (Hughes et al., 2004, 2011). This could indicate that this physiological process is involved in response to the conscious action of mental imagery.

## 6 From oculomotor integrator to attractor network, working memory, and consciousness

By contrast with consciousness research, advances in the field of motor control, and particularly oculomotor research, demonstrate a different path forward. Starting from precise scientific facts, such as the eye saccade, animal studies measuring motoneuron electrical signals have shown that two types of signals underline this rapid movement and its subsequent maintenance: one proportional to velocity, followed by another proportional to position. On this basis, Robinson (1968, 1989) proposed a theoretical model suggesting that a neural network must perform a mathematical integration of the velocity signal generated by neurons in the reticular formation to produce the position signal responsible for gaze holding. It was only about two decades later that the oculomotor integrator was identified in the prepositus nuclei of the brainstem (Cheron et al., 1986a; Cheron et al., 1986b; Cannon and Robinson, 1987), and it was demonstrated that neurons in these nuclei indeed perform a mathematical integration of their input signals (Godaux and Cheron, 1993, 1996). This fundamental operation has also been shown to rely on recurrent networks (Draye et al., 1996, 1997). Prepositus neurons studied *in vitro* have shown endogenous beta–gamma oscillatory activity that likely contributes to the integration function (Idoux et al., 2006) by facilitating the network recurrence. Similarly, the presence of NMDA synapses within this network increases the time constants of synaptic currents by a factor of 100, allowing the tonic signal required for position holding to persist longer in the absence of corrective feedback. Injection of an NMDA receptor blocker into the integrator abolishes the position signal (Cheron et al., 1992; Mettens et al., 1994). Based on these oculomotor experiments, one could suggest that the neuronal mechanisms involved in maintaining eye position in space may be transposed to those underlying not only the stabilization of other body segments, but also cognitive domains, such as the maintenance of an idea or the sustained intention to move (Cheron, 2020). The concept of a neuronal integrator has been proposed as a mechanism for working memory (McCormick, 2001), and it may also contribute to processes underlying conscious awareness. We may propose that the neural dynamics involved in maintaining a limb’s position could be translated into the cognitive processes involved in maintaining an idea (Cheron et al., 2023). Given that neuronal integrators rely on locally organized recurrent networks, such as within each prepositus nucleus on the left and right sides of the brainstem (Cheron et al., 1986a; Cheron et al., 1986b; Delgado-García et al., 1989), where reverberating cascades ultimately generate the final position signal, it is reasonable to assume that similar integration processes operate within the recurrent architectures proposed by the theory of local recurrency (Lamme and Roelfsema, 2000; Lamme, 2020). According to this framework, consciousness emerges from reentrant cortical loops that enhance and stabilize learning processes. In this line, Cleeremans et al. (2020) propose the existence of a common prediction-driven learning mechanisms, perceptual and self-awareness, operating over three loops: an inner loop, a perception–action loop, and a self–other loop.

Models derived from experiments on the oculomotor integrator have paved the way for a better understanding of the dynamics of neuronal populations and the architecture of their circuits (Seung, 1996; Aksay et al., 2000, 2001; Seung et al., 2000). This allows us to consider the transition from the notion of an integrator to that of an attractor (Khona and Fiete, 2022). The concept of an attractor is central to dynamical systems and their related states. A dynamical system corresponds to a set of variables whose values change over time. The state of the system is a vector point in state space, and the attractor is the minimal energy state toward which neighboring states converge (Hopfield, 1982, 1984). In this sense, the oculomotor integrator is regarded as a continuous attractor (line attractor) (Khona and Fiete, 2022).

Recently, Klatzmann et al. (2025) developed a neuronal model based on the macaque brain connectome, proposing the existence of a dynamic bifurcation leading to the ignition processes (Fisch et al., 2009; Moutard et al., 2015) of sensory consciousness. This study is grounded on neuronal units with realistic biophysical constraints and the presence of AMPA receptors capable of encoding fast events as 50-ms pulses, reaching a neuronal firing peak of approximately 60 Hz originating in the primary visual cortex (V1). From there, this pulse signal propagates through a large-scale network via NMDA synapses until it reaches the prefrontal cortex. This signal, in a manner similar to the velocity pulse that generates saccadic eye movements, is mathematically integrated within the dynamic network so that a tonic signal gradually builds up over time, reaching a stable plateau of around 30 Hz at approximately 250 ms and persisting beyond 400 ms. It is interesting to mention that the time of arrival at the plateau fits relatively well with the P300 wave emergency and the 270 ms bifurcation point on evoked potentials trace measured during the attentional blink indicating conscious recognition of a sensory event (Sergent et al., 2005). The Klatzmann et al.’s model further suggests that visual information becomes consciously perceived only when this bifurcation successfully occurs, that is, when the integration process within recurrent neural loops is achieved. These loops include inhibitory (GABAergic) synapses that play a crucial role in preventing information overflow, a mechanism previously identified in models of the oculomotor integrator (Draye et al., 1996; Arnold and Robinson, 1997).

## 7 Conclusion

In conclusion, although the authors did not mention this analogy between their ignition model and data from the oculomotor integrator, responsible for generating and maintaining eye position in space, it seems important to highlight this parallel, given the critical role of NMDA receptors. Pharmacological blockades of these receptors produce major impairments in eye position maintenance (Cheron et al., 1992; Mettens et al., 1994) and, by logical extension, should prevent consciousness ignition according to Klatzmann et al. (2025) model. This again underscores that sensory information processing and motor production share common neurophysiological mechanisms, emphasizing the importance of promoting approaches that combine sensory perception and attentional processes during various motor tasks. In addition, the introduction of neural integrator concept into machine learning models using EMG signals has enhanced their performances, leading to better myoelectric prosthesis control (Simar et al., 2024).

## Data Availability

The original contributions presented in the study are included in the article/supplementary material, further inquiries can be directed to the corresponding author.
